# Real-time cine first-pass perfusion imaging enables rapid detection of functionally significant high-grade coronary stenosis

**DOI:** 10.1186/1532-429X-17-S1-O12

**Published:** 2015-02-03

**Authors:** Behzad Sharif, Reza Arsanjani, Hsin-Jung Yang, Rohan Dharmakumar, Noel Bairey C Merz, Daniel S Berman, Debiao Li

**Affiliations:** 1 Biomedical Imaging Research Institute, Cedars-Sinai Medical Center, Los Angeles, CA, USA; 2 Cedars-Sinai Heart Institute, Los Angeles, CA, USA

## Background

Among the spectrum of patients with coronary artery disease (CAD), those suffering from high-grade stenosis are at a significantly elevated risk for adverse events [1]. Such high-grade disease may result in the so-called "coronary steal" phenomenon under vasodilator stress [2] thereby inducing wall-motion abnormalities (WMAs) in the affected territories, referred to as transient ischemic dilation. We developed a "real time" cine first-pass perfusion (FPP) CMR method for concurrent imaging of myocardial function and perfusion. We hypothesized that this method is capable of simultaneously capturing stress-induced perfusion defects and WMAs in a single ungated scan.

## Methods

Canines (n=9) with surgically implemented reversible stenosis below the first diagonal along the left anterior descending (LAD) artery (≈90% stenosis) were studied at 3T. Real-time adenosine stress/rest cine perfusion data was acquired using an ungated continuously-sampled FPP sequence without saturation recovery preparation [3,4]. The T1-weighted acquisition scheme used a steady-state FLASH sequence *with 3-5 slice coverage* and no time-gap in between consecutive slice-interleaved radial readouts, acquiring 5,000 projections per slice during the 40-second real-time FPP scan (flip: 30°, echo spacing: 2.7 ms, in-plane resolution: 1.4x1.4 mm^2^). Retrospective cardiac self-gating was performed automatically using a high temporal-resolution reconstruction of the mid slice at low spatial resolution. The self-gating information was then used to perform a cardiac phase-resolved reconstruction of the FPP data for all slices at high resolution employing a compressed sensing approach. WM for the cine perfusion images was scored on a standard 1-4 scale. To test the clinical feasibility of this approach, patients (n=3) with known high-grade (≥90%) right coronary artery (RCA) stenosis were studied.

## Results

**Figure 1:**
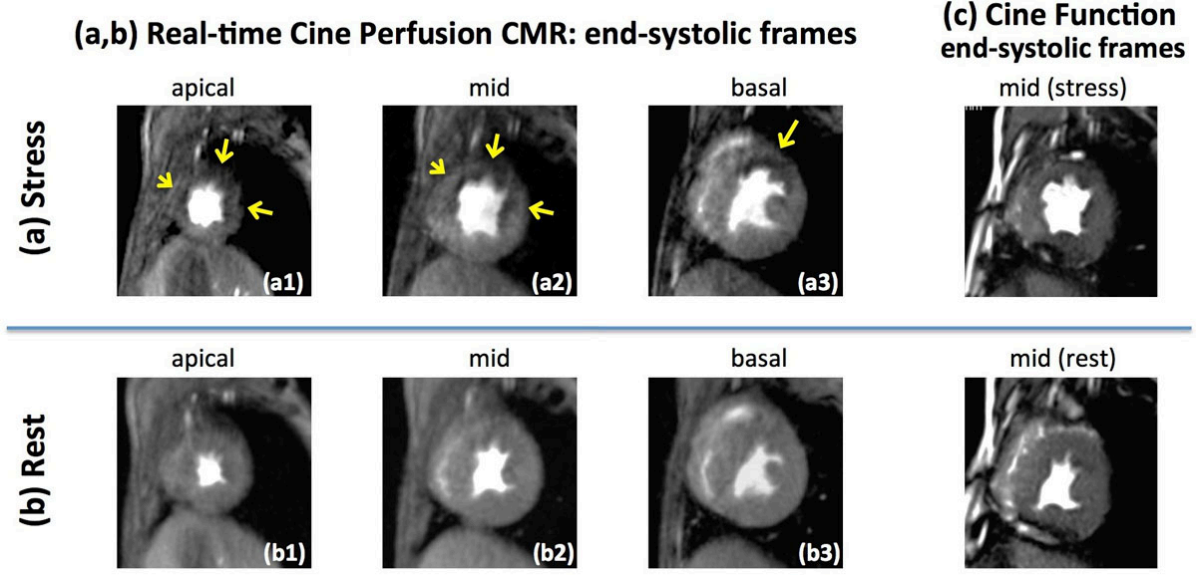
Representative images from the ischemic canine stress/rest studies (n=9) with high-grade LAD stenosis. (a,b): End-systolic adenosine stress and rest first-pass perfusion images (peak enhancement phase) using the developed real-time cine FPP technique. All of the stress-induced perfusion defect territories (arrows) show WMAs compared to the rest scan (larger end-systolic LV cavity area at stress compared to rest), consistent with the coronary steal (ischemic dilation) phenomenon. (c): standard SSFP cine end-systolic images (mid slice), showing a similar stress-versus-rest WMA pattern as in (a,b). This confirms that the real-time cine perfusion technique was capable of accurately capturing the wall motion at stress (i.e., achieving sufficiently high temporal resolution).

**Figure 2:**
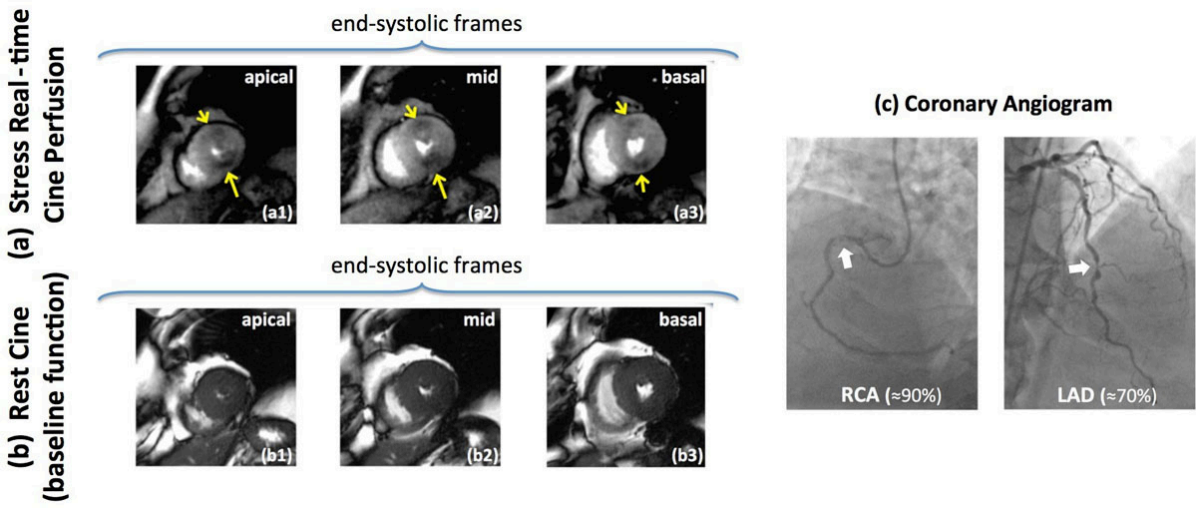
Representative CAD patient study with high-grade RCA stenosis. (a): End-systolic frames (peak myocardial enhancement phase) corresponding to the proposed real-time cine first-pass perfusion method at the apical, mid, and basal ventricular levels. (b): standard SSFP cine scan (end-systolic frames) in the same 3 slices performed at baseline (resting state) prior to adenosine infusion. (c): invasive coronary angiogram. The observed stress perfusion defects (yellow arrows) are consistent with the angiogram in (c), which shows a high-grade stenosis (≈90%) in proximal RCA (dominant vessel) and ≈70% stenosis in mid LAD. For the mid slice, the perfusion defect territory (inferior wall) shows WMA in comparison to the baseline end-systolic frame, i.e., a larger end-systolic LV cavity area is seen in (a2) compared to (b2). This is consistent with the angiographically documented high-grade RCA stenosis.

Real-time stress FPP scans in stenotic dogs (Fig. 1) showed worsening of WM in the perfusion defect territories compared to resting function, consistent with ischemic dilation (slice-averaged stress WM score for defect region: 2.8 vs. 1.6 for rest; p < 0.05). The close agreement of WMA vs. standard cine (Fig. 1) indicates the high temporal resolution of the real-time method. The mid-slice inferior perfusion defect in the example patient study (Fig. 2) coincides with the WMA (larger LV cavity area) compared to rest function, consistent with the angiogram. The other 2 patients showed similar results.

## Conclusions

We presented a multi-slice *cine* FPP method capable of *simultaneous* detection of stress-induced perfusion defects and WMAs in a *single* ungated scan using continuous acquisition (<1 minute). Our initial results demonstrate that worsening of WM (compared to rest) in the perfusion defect territories seen in the real-time stress cine FPP scan may be a marker of severe CAD.

## Funding

American Heart Association Scientist Development Grant 14SDG20480123; NIH National Heart, Lung and Blood Institute grant nos. K99 HL124323-01, R01 HL038698-18, R01 HL091989-05, R01 HL090957-01.
